# Charges against a Surgeon

**Published:** 1861-08

**Authors:** 


### CHARGES AGAINST A SURGEON.

The Burlington (Vt.) *Free Press* publishes the affidavits of two members of the 1st Vermont Regiment, stating that the treatment of members of the Regiment, when sick, by Surgeon Sanborn, was harsh and brutal, and that he was often so intoxicated as to unfit him for the performance of his duties.

Lieut. Col. Washburn says :—Dr. Sanborn has had many difficulties to contend with. He has been compelled to organize a Hospital many times without sufficient room or conveniences ; there has been a great deal of sickness in our camp ; frequently at the morning report of the sick, a large number have been awaiting his examination ; many times since we came here the Hospital has been entirely filled, and no room—and he would have been more than human if at times, he had not found his patience exhausted. But he has so far as my observation extends, endeavored faithfully to perform his duty. The Surgeon, Dr. Sanborn, and Assistant Surgeon, Dr. Child, are attentive, careful and earnest, and the people have no need of feeling anxious that their friends are not well cared for.

